# A Dead Philanthropist

**Published:** 1911-04-29

**Authors:** 


### A DEAD PHILANTHROPIST.

### MR. PASSMORE EDWARDS' HOSPITAL FOUNDATIONS.

Till Mr. Macdonald published his catalogue in ninety-three pages of the Passmore Edwards institutions, it was hardly realised how many hospitals and convalescent homes Mr. Edwards had established. The mere list of them, however, is enough to show that Mr. Passmore Edwards, whose death last Sunday, at the age of 88, removed so famous a philanthropist, did not confine himself to the foundation of public libraries, of educational institutions, of the busts of statesmen, or of the drinking-fountains with which he is associated in the public mind. Indeed, it has been sometimes suggested that he wished to found as many cottage hospitals as there are letters in his name. Whether that were so or not, at the present moment Liskeard, Acton, Wood Green, Tilbury, and Willesden owe their cottage hospitals to him. It may be said, in short, that Passmore Edwards was not only a supporter of the voluntary hospital system, but also had his own ideas about its future, as was shown in the particular encouragement which he gave to the cottage hospital and the convalescent home, both new movements in his day.

His life and work in the general sense have been chronicled abundantly elsewhere, and we need only recall the close association of his name with late Victorian journalism, especially with the most successful period of the *Echo's* existence. His wealth was due to the prosperity of the numerous periodicals which at one time or another were founded or purchased by him. A Cornishman by birth, the son of a carpenter, his career began at eighteen in a London publisher's office, was chequered by illness and bankruptcy, and vindicated at last by a dinner from his creditors, whom he paid with generous interest. Afterwards came the prosperity of the newspaper proprietor. But, it should be remembered, this success was delayed till 1872, when he was nearly fifty years of age. Once wealth was in his grasp, health and education were the principal objects which he determined to encourage, and the very keen sense of business which had established his future is aptly shown by the vitality of his foundations.

One fact about Mr. Passmore Edwards' work for the hospitals is characteristic of his methods and aims, and that is his independence. When once a cottage hospital or a convalescent home had been founded by him, he did not interfere. His wishes and his keen eye for business laid down the lines of development, and these were determined in his deed of gift. That done, his foundation began an independent existence. Consequently, the posts which he was induced to accept were few and honorary, the chief among them being his vice-presidency of Charing Cross Hospital and the life governorships of the Westminster and Metropolitan Hospitals.

The Charing Cross Hospital owes to him its convalescent home at Limpsfield, Surrey, which was opened in 1896 by the late King. To it Mr. Edwards gave £5,000, and as his generosity supplied the money, so it was his energy which solved all the difficulties. Among them was the choice of a site, which greatly exercised the minds of the Committee and of the landlords, who, when they knew for what the land was wanted, resolutely refused to sell. Only by concealing his personality was the land acquired at last, and kept, in spite of every offer for repurchase. The early nineties were busy with his hospital foundations, the first of which was, we believe, the Home at Perranporth, founded in connection with the Royal Cornwall Infirmary, Truro. In 1893 the Willesden Cottage Hospital was opened, to supply the needs of a population which had no other hospital within three miles. In this case Passmore Edwards came to the rescue of a committee which was unable to pay the contractor, though the building was practically finished. Wood Green hereupon appealed to the rescuer, who said at the laying of the foundation-stone that he was willing to provide "a series of cottage hospitals round London where they were required and would be maintained." The hospital was opened in 1895. During the same year the Caxton Convalescent Home for men employed in the printing and allied trades was opened, Limpsfield again being chosen. Mr. Edwards did not perform the opening ceremony, on the characterisitic ground that it might injure its prospects as a self-supporting institution if he did. In the meantime, 1894, Falmouth and Penrhyn had been supplied with an institution which for three years had proved unobtainable locally.

Tilbury next claimed the philanthropist's attention, and in 1896 the dock population was spared the necessity of being carried, in case of accident, to Gravesend or to Poplar, by the hospital which he supplied. Here, again, for twelve years previously local effort had accomplished nothing. At the opening ceremony he emphasised the need for the better housing of the working classes, taking the Tilbury district as a text of what to avoid. This year, 1896, the Cottage Hospital at Liskeard was also opened, being, as it were, complementary in aim to that previously established at Falmouth. Mr. Edwards remarked at the opening that "it was the first instance within his experience or memory where the civic authority had become the custodians, in the public interest, of an institution of the kind," the Corporation having accepted the gift of the hospital from him.

In 1897 Princess Louise opened at Cranbrook the Convalescent Home, which Mr. Edwards gave in order that the Metropolitan Hospital might use the Spicer bequest of £10,000 for the maintenance of a home, which was badly needed, but which Mr. Spicer's will precluded it from building with his money.

Such in outline was Mr. Passmore Edwards' work for hospital development, and we do not think a better instance of the practical good he effected could be given than that of his saving the Spicer bequest for the Metropolitan Hospital. His aim was always to help institutions or localities to help themselves. That is why he gave rather than maintained his hospitals, and why, no doubt, he left their management to local public spirit, without interference from him.

